# Molecular mechanisms of NLRP3 inflammasome activation

**DOI:** 10.1038/s12276-026-01656-9

**Published:** 2026-03-25

**Authors:** Hyo Jung Shin, In Soo Kim, Jin Kyung Kim, Eun-Kyeong Jo

**Affiliations:** 1 Department of Biochemistry and Cell Biology, Eulji University School of Medicine, Daejeon, Republic of Korea; 2 Department of Medical Science, Chungnam National University College of Medicine, Daejeon, Republic of Korea; 3 Department of Pharmacology, Chungnam National University College of Medicine, Daejeon, Republic of Korea; 4 Biomedical Research Institute, Chungnam National University Hospital, Daejeon, Republic of Korea; 5 Department of Microbiology, Keimyung University School of Medicine, Daegu, Republic of Korea; 6 Department of Microbiology, Chungnam National University College of Medicine, Daejeon, Republic of Korea

**Keywords:** Inflammasome, Cell death and immune response

## Abstract

The NOD-like receptor protein 3 (NLRP3) inflammasome is among the most extensively studied multiprotein complexes, driving the maturation of pro-interleukin-1β (pro-IL-1β) and pro-IL-18—into their active forms, IL-1β and IL-18, respectively. The activation of the NLRP3 inflammasome is a multifaceted process triggered by a diverse array of stimuli, including pathogens, environmental particles and endogenous stress signals. Previously, NLRP3 inflammasome activation was considered a straightforward two-step process: signal 1, which induces the expression of NLRP3 and proinflammatory cytokines, and signal 2, which promotes the assembly of the inflammasome complex through mechanisms such as ionic fluxes, mitochondrial dysfunction and lysosomal damage. However, more intricate mechanisms have now been elucidated, particularly regarding the ‘priming’ step, involving the regulation of its post-translational modifications. Recent studies have comprehensively identified the core components of the NLRP3 inflammasome complex, its interacting complex partners, and regulatory mechanisms. Here we delve into the current understanding of the NLRP3 inflammasome activation mechanisms and explore its regulatory networks. Enhanced insights into the molecular and signaling pathways controlling this specialized inflammasome activation may pave the way for novel applications of NLRP3 inflammasome regulation to advance human health and prevent numerous diseases linked to the NLRP3 inflammasome.

## Insights into NLRP3 inflammasome activation and regulation

Inflammasomes are important components of our innate immune system that help detect harmful substances and trigger inflammation to protect us. The NLRP3 inflammasome is a well-studied type that plays a key role in this process. However, when it does not function properly, it can lead to diseases. Here researchers explored how small changes in proteins, called post-translational modifications (PTMs), affect the NLRP3 inflammasome. The study reviewed various PTMs, such as phosphorylation, ubiquitination and others. These modifications can alter how the inflammasome works by affecting its assembly, stability and interactions with other proteins. The researchers used existing studies to better understand these processes. They found that PTMs are crucial for controlling NLRP3 inflammasome activity. This understanding could help develop new treatments for diseases caused by improper inflammasome activation.

This summary was initially drafted using artificial intelligence, then revised and fact-checked by the author.

## Introduction

Multiprotein oligomeric complexes consist of several components, including a pattern-recognition receptor, an adapter and an effector (caspase-1). Inflammasomes are crucial sensors for signals from various pathogen-associated molecular patterns (PAMPs) and danger-associated molecular patterns (DAMPs). Their assembly triggers the activation of inflammatory caspases, gasdermin cleavage-mediated pyroptosis and the release of proinflammatory cytokines^1–3^. Among these, NOD-like receptor protein 3 (NLRP3) inflammasome is extensively studied^2,3^. Activation of the NLRP3 inflammasome recruits and activates caspase-1, facilitating the cleavage and secretion of interleukin (IL)-1β and IL-18, while also triggering gasdermin D cleavage-induced proptosis^1–4^.

The canonical pathways of priming, assembly and proptosis are well-established in the context of NLRP3 inflammasome activation. Recent research has increasingly focused on the role of post-translational modifications (PTMs) in regulating inflammasome activity at the molecular level. Various PTMs, such as phosphorylation, ubiquitination, SUMoylation, acetylation and glycosylation, can modulate inflammasome components by influencing protein–protein interactions, stability and localization^5^.

The activation of the NLRP3 inflammasome results in the production of inflammatory cytokines IL-1β and IL-18, which signal inflammation, recruit and activate immune cells, and induce gasdermin-mediated pyroptotic cell death. These processes are vital for neutralizing or eliminating infectious threats, promoting immune defense and maintaining cellular homeostasis against infection and inflammation^1–3,6^. However, dysregulated inflammasome activation is linked to numerous inflammatory, autoimmune and degenerative diseases. Emerging evidence suggests that aberrant PTMs of inflammasome components contribute to this dysregulation^7,8^. This Review delves into PTMs in the NLRP3 inflammasome pathway, their molecular mechanisms and functional consequences for the signaling cascade and highlights key questions about the intricate regulatory network of molecular signals involved.

## Overview of the structure of the NLRP3 inflammasome complex

**Fig. 1:**
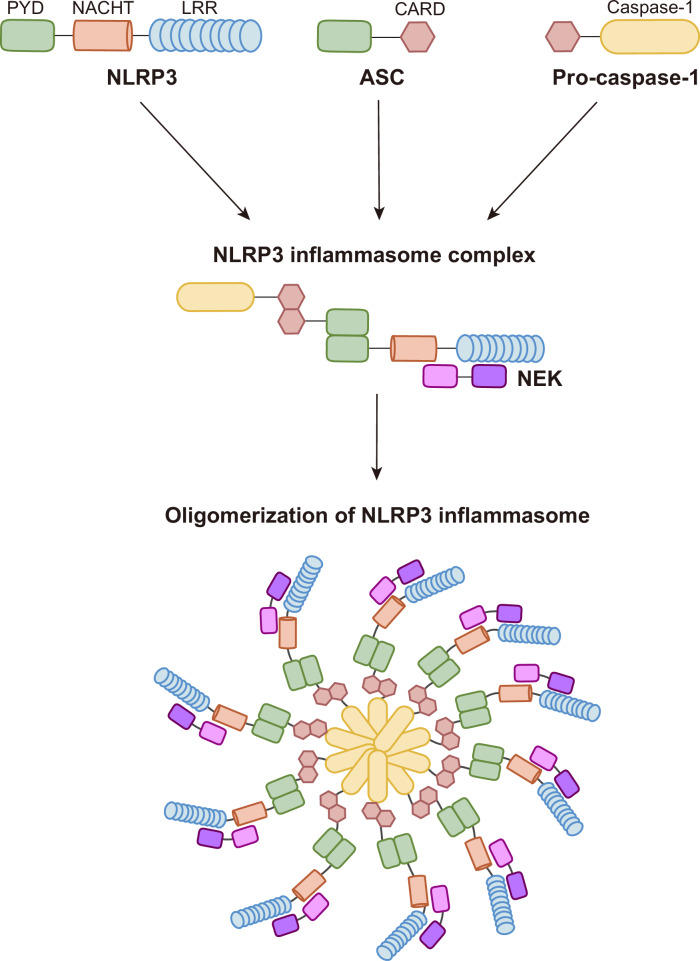
Structural organization and assembly of the NLRP3 inflammasome. A schematic representation of the NLRP3 inflammasome complex. NLRP3 is composed of an N-terminal PYD, a central NACHT domain and C-terminal LRR domains. Upon activation, NLRP3 undergoes oligomerization and recruits the adapter protein ASC via PYD–PYD interactions. ASC subsequently engages pro-caspase-1 through CARD–CARD interactions, leading to the assembly of the NLRP3 inflammasome and the activation of caspase-1. NEK7 is depicted as an essential licensing factor that interacts with the NACHT domain of NLRP3 to facilitate inflammasome assembly.

The structure of NLRP3 comprises three main domains, each playing a vital role in its function as part of the inflammasome. A detailed overview of its structure is shown in Fig. 1.

The first domain is the PYD, located in the N-terminal region of NLRP3. This domain facilitates protein–protein interactions, particularly in inflammasome formation. The PYD of NLRP3 interacts with the PYD of the adapter protein apoptosis-related speck-like protein containing a caspase recruitment domain (ASC), which is critical for ASC to assemble the inflammasome complex. The second domain is the NACHT domain (nucleotide-binding and oligomerization domain). Centrally positioned within NLRP3, the NACHT domain is essential for ATP binding and hydrolysis, promoting the oligomerization of NLRP3, a crucial step in inflammasome activation. It includes several subdomains: the nucleotide-binding domain, helical domain 1 (HD1), winged-helix domain and HD2. Last, the LRR domain forms the C-terminal region of NLRP3. This domain is implicated in autoinhibition, keeping NLRP3 inactive under normal conditions while also sensing diverse danger signals, such as microbial motifs, environmental irritants and endogenous stress signals.

ASC comprises an N-terminal PYD and a C-terminal caspase recruitment domain (CARD). It interacts with NLRP3 via homotypic PYD–PYD interactions. The resulting prion-like ASC filament serves as a platform for recruiting effector pro-caspase-1 through CARD–CARD interactions^3,9–11^. Within this complex, centrosomal NIMA-related kinase 7 (NEK7), by interacting with NLRP3 and ASC, plays a critical role in assembling the NLRP3 inflammasome disc, which recruits caspase-1 (refs.^4,10,12,13^). Recently, structural analysis revealed that the NLRP3 oligomeric cage in the trans-Golgi network (TGN) disperses vesicles toward the MTOC. At this site, NLRP3 binds to NEK7, which opens the cage structure and rearranges NLRP3 into an ‘active disc’ shape, thereby promoting NLRP3–ASC interaction and inflammasome complex assembly via a hybrid PYD–PYD and CARD–CARD filamentous scaffold formation^4,12^. These structural insights have advanced our understanding of the complex NLRP3 activation mechanism. Further studies will illuminate the precise structural events occurring during inflammasome activation under various stresses and stimuli. Understanding these mechanisms will aid in designing specific inhibitors or activators to target key steps in NLRP3 inflammasome assembly, enabling selective modulation of inflammasome-related disease pathologies.

## Two signals of NLRP3 inflammasome activation

**Fig. 2:**
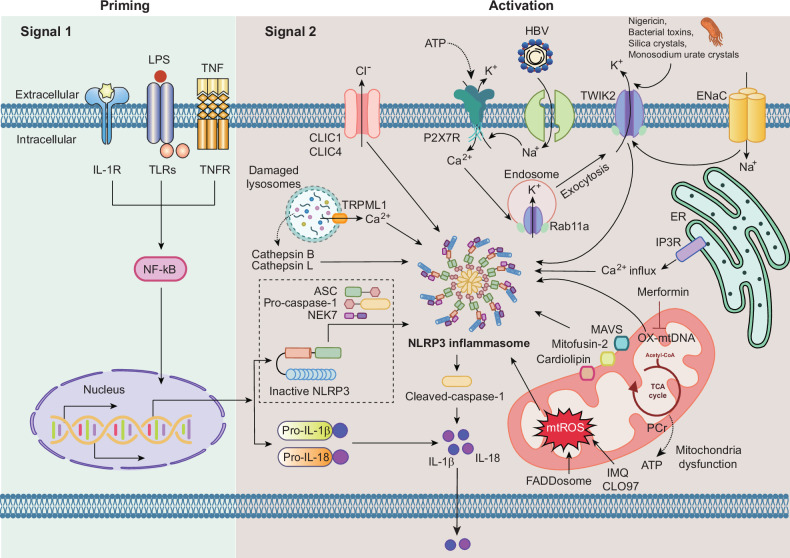
Molecular mechanisms underlying priming and activation of the NLRP3 inflammasome. A detailed schematic illustrating the two-step process required for NLRP3 inflammasome activation and the diverse upstream signals that converge on this pathway. The priming step (signal 1) is initiated by PAMPs, DAMPs or inflammatory cytokines that activate TLRs, IL-1 receptor (IL-1R) or TNF receptor (TNFR). These signals induce NF-κB activation, resulting in transcriptional upregulation of inactive NLRP3 as well as the pro-forms of the inflammatory cytokines IL-1β and IL-18. Priming also licenses NLRP3 through post-translational and metabolic reprogramming events. The activation step (signal 2) is triggered by a wide range of extracellular and intracellular stimuli, including extracellular ATP-mediated activation of the P2X7 receptor and associated K⁺ efflux, pore formation by TWIK2 and ENaC, and dysregulated Na⁺ and Ca²⁺ influx. Additional activation signals include mitochondrial dysfunction characterized by increased mtROS production, oxidized mitochondrial DNA (OX-mtDNA) release, cardiolipin externalization, and altered mitochondrial dynamics involving MAVS and mitofusin-2. ER–mitochondria Ca²⁺ transfer via the IP3R, lysosomal destabilization with cathepsin B and L release and endosomal stress further contribute to NLRP3 activation. Crystalline and microbial stimuli, such as nigericin, bacterial toxins, silica crystals and monosodium urate crystals, are also depicted as potent activators. Metabolic and trafficking pathways modulate inflammasome activation, including TCA cycle-derived metabolites, acetyl-CoA availability, PCr, Rab11a-dependent vesicular trafficking and ion channel regulators such as CLIC1, CLIC4 and TRPML1. Pharmacological modulators, including metformin, are shown to interfere with mitochondrial and metabolic signals that restrain NLRP3 activation. Viral components, including HBV, are illustrated as upstream modulators of inflammasome signaling.

Canonical NLRP3 inflammasome activation occurs through two distinct steps: signal 1 (priming phase) and signal 2 (activation phase) (Fig. 2). The priming phase is initiated by toll-like receptors (TLRs) or cytokine receptors in response to PAMPs or inflammatory cytokines such as TNF. These signals ultimately enhance NLRP3 protein expression and promote the production of pro-IL-1β and pro-IL-18. In addition, this priming step is tightly regulated by PTMs of the NLRP3 inflammasome components, effectively ‘licensing’ NLRP3 and related molecules to respond to various activation signals. These licensing mechanisms will be explored in detail in the following section.

During the ‘activation’ phase, a range of chemically and structurally distinct stimuli, such as adenosine triphosphate (ATP)^14^, nigericin^15,16^, bacterial toxins^17,18^ and particles such as silica crystals^19,20^ and monosodium urate crystals^21^ have been identified. These diverse signals potently drive NLRP3 inflammasome assembly and caspase-1 cleavage and activation, resulting in the maturation and secretion of IL-1β and IL-18. However, how these varied stimuli induce a shared cellular response during the second step of NLRP3 inflammasome activation remains unclear. So far, several specific cellular events, at least three crucial mechanisms, have been identified as essential to initiating NLRP3 inflammasome complex assembly and activation. In this section, we briefly outline the current understanding of three primary mechanisms (ionic flux, inhibition of the mitochondrial electron transport chain and lysosomal damage) along with several other pathways of second signal activation^22^.

### Ionic flux

The movement of several ions—potassium (K^+^), sodium (Na^+^), chloride (Cl^−^) and calcium (Ca^2+^)—across the cell membrane generates signal 2, which triggers the assembly and activation of the inflammasome complex. A landmark study by the Núñez group reported that the most consistent mechanism for signal 2 in NLRP3 inflammasome activation involves K⁺ ion permeation through the cell membrane, known as ‘K⁺ efflux’. Common NLRP3-activating stimuli, including ATP, bacterial toxins and environmental particles, initiate inflammasome activation via this K⁺ efflux mechanism^23^. Recent research has uncovered molecular mechanisms by which various ionic fluxes contribute to NLRP3 activation. The two-pore domain K^+^ channel TWIK2 has been identified as the potassium efflux channel driving NLRP3 activation^24^. In addition, endosomal TWIK2 fuses with and translocate to the plasmalemma, a process regulated by Rab11a for downstream inflammasome activation^25^. However, exceptions exist where intracellular potassium levels are not reduced. For instance, imiquimod and CL097 can activate the NLRP3 inflammasome without lowering potassium levels^26^. In human monocytes, lipopolysaccharide stimulation directly activates the NLRP3 inflammasome through a noncanonical pathway without inducing K⁺ efflux^27^.

A decreased extracellular Na^+^ concentration can suppress NLRP3 activation, though it is not strictly required for NLRP3 modulation. Interestingly, simply increasing intracellular Na^+^ levels is insufficient to activate NLRP3^23^. For example, monosodium urate crystals, after fusing with acidic lysosomes, elevate intracellular Na^+^ levels, causing water influx that creates a low-potassium environment, ultimately activating NLRP3^28^. In addition, a recent study demonstrated that hepatitis B virus (HBV) induces potassium efflux-dependent NLRP3 activation, which is amplified by intrahepatic danger signals through increased sodium influx into macrophages^29^. Thus, while Na^+^ flux’s role in inflammasome activation appears indirect, it may synergize with and relate to K^+^- associated signals in a context-dependent manner.

The involvement of chloride (Cl^−^) in NLRP3 activation remains controversial. Certain chloride channels, such as chloride intracellular channels (CLICs), have been shown to promote the NLRP3–NEK7 interaction downstream of K⁺ efflux and mitochondrial reactive oxygen species (mtROS)^30^. Both CLIC1 and CLIC4, which are specific chloride channels, contribute to the transcriptional activation of IL-1β during the priming step of NLRP3 inflammasome activation^31^. Furthermore, mutations in the cystic fibrosis transmembrane conductance regulator (CFTR), observed in cystic fibrosis patients, result in defective CFTR-mediated chloride and bicarbonate transport, which is linked to dysregulation of epithelial sodium channels (ENaC). A recent study demonstrated that ENaC-mediated sodium influx causes exaggerated and dysregulated inflammatory responses in cystic fibrosis patients^32^. In addition, WNK (with no lysine (K)) serine/threonine kinases have been identified as negative regulators of NLRP3 activation by modulating SLC12A2, a Cl^−^/cation cotransporter that also influences potassium levels^33^. These findings suggest a connection between Cl^2+^, sodium and K⁺ fluxes in modulating NLRP3 inflammasome activation. Further research is necessary to clarify the roles of ion transporters and the detailed molecular mechanisms underlying Cl^2+^ flux, as well as its cooperation with Na^+^ flux in signal 2 activation or inhibition.

The role of Ca^2+^ in response to various stimuli has also been documented^34^. However, it remains widely debated, and several models have been proposed. One study reported that NLRP3 activators induce Ca^2+^ influx via the IP₃ receptor (IP3R), leading to NLRP3 activation, while intracellular Ca^2+^ triggers spontaneous inflammasome activation in cells of patients with gain-of-function mutations in NLRP3, such as those in cryopyrin-associated periodic syndromes^35^. Excessive intracellular calcium can damage mitochondria and mitochondrial dysfunction may subsequently trigger NLRP3 activation^34^. Nevertheless, Ca^2+^ signaling does not consistently result in NLRP3 inflammasome activation and does not appear to serve as a universal trigger. Collectively, while K^+^ efflux is the most critical signal, it is not the sole determinant of NLRP3 inflammasome activation. Future structural and functional studies are needed to elucidate the complex mechanisms through which each ion flux collectively influences the regulation and coordination of NLRP3 inflammasome assembly.

### Mitochondria and the respiratory chain

Although still debated, it is thought mitochondria are to serve as a platform for the assembly of the NLRP3 inflammasome. The generation of mtROS from dysfunctional mitochondria or inhibition of the mitochondrial respiratory chain can trigger NLRP3 inflammasome assembly^36,37^. In addition, mitochondrial dysfunction caused by autophagy defects leads to the translocation of mitochondrial DNA (mtDNA), directly activating the NLRP3 inflammasome^37,38^. Furthermore, TLR signaling induces the oxidized form of mtDNA, which is critically required for NLRP3 inflammasome activation^39^. Recent studies have shown that metformin, a drug used to treat diabetes, alleviates acute respiratory distress syndrome (ARDS) pathology by inhibiting mtDNA synthesis, suppressing oxidized forms, improving NLRP3 inflammasome activation, and reducing IL-1β production^40^.

However, the physiological role of mitochondrial dysfunction and mtROS production in NLRP3 inflammasome activation remains uncertain. A prior study suggests that a drop in cytosolic K⁺, rather than mtROS production, is critical for NLRP3 inflammasome activation^23^. More recently, a ROS-independent mechanism was proposed for NLRP3 inflammasome activation. Inhibitors of mitochondrial electron transport chain complexes I, II, III and V were found to prevent NLRP3 inflammasome activation. Metabolomic analysis identified phosphocreatine (PCr), which sustains ATP levels, as essential for this process^41^. By contrast, in a gastric mucosal injury model, FADDosome-mediated mitochondrial dysfunction, altered glycolysis and enhanced mtROS production were implicated in NLRP3 inflammasome activation and pathologies associated with portal hypertensive gastropathy^42^. These findings indicate that pathological conditions characterized by mtROS hyperactivation and mitochondrial damage promote NLRP3 inflammasome activation. Further research is required to clarify which mitochondrial components mtROS or mtDNA contribute to NLRP3 inflammasome activation across various cell types and pathological conditions.

Moreover, specific mitochondrial proteins such as mitofusin-2, cardiolipin and mitochondrial antiviral signaling protein (MAVS) have been shown to facilitate the localization of NLRP3 in mitochondria or its binding to NLRP3, driving the assembly of the NLRP3 complex^43–45^. In this Review, we do not delve into the detailed mechanisms by which mitochondrial components activate the NLRP3 inflammasome.

### Lysosomal damage

Studies have shown that particulate matter, including monosodium urate, alum, asbestos, silica, cholesterol crystals and amyloid-β, can activate the NLRP3 inflammasome complex^1–3,19,46,47^. Several mechanisms, such as lysosomal acidification followed by Na^+^ release, which triggers water influx and K^+^ efflux, are involved in lysosomal damage-associated inflammasome activation^19,28^. The release of active lysosomal enzymes, such as cathepsin B, into the cytosol may also contribute to NLRP3 inflammasome activation during the phagocytosis of particulate matter^19,48^. Furthermore, either cathepsin B or cathepsin L is necessary for NLRP3 inflammasome priming and activation induced by cholesterol crystallization in modified low-density lipoprotein (LDL)^49^. A recent study using small-molecule library screening to identify NLRP3 agonists discovered that a lysosomal disruptor triggers transient receptor potential mucolipin 1 (TRPML1)-dependent Ca^2+^ flux in lysosomes, which is followed by mitochondrial damage and leads to NLRP3 inflammasome activation^50^.

These findings suggest that different NLRP3 stimuli may employ multiple mechanisms simultaneously to induce NLRP3 inflammasome assembly. For instance, in the context of extracellular ATP signals, the P2X7 receptor acts as a purinergic receptor and functions as an ATP-gated cation channel, enabling Na^+^ and Ca^2+^ fluxes across cells, along with K^+^ efflux^51,52^. However, questions remain about how various NLRP3 stimuli converge on shared or intermediary pathways to activate NLRP3 inflammasome assembly across different cell types and disease settings.

### Immunometabolism

**Fig. 3:**
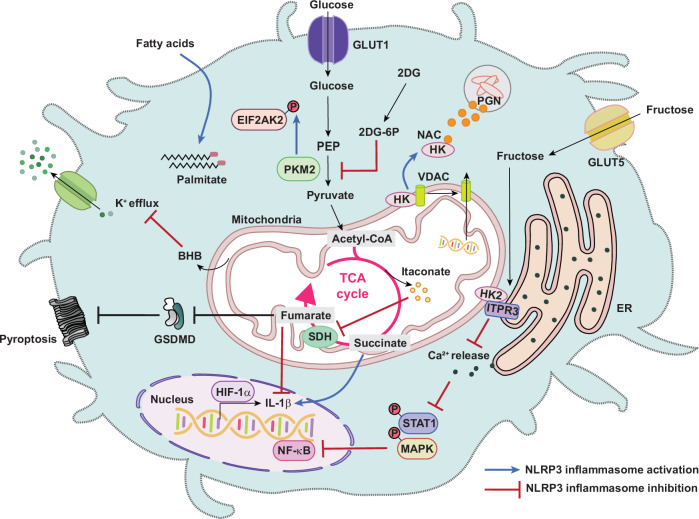
Metabolic regulation of NLRP3 inflammasome activation and inhibition. A schematic illustration depicting the metabolic pathways and signaling nodes that regulate NLRP3 inflammasome activation or suppression. Enhanced glucose uptake through glucose transporters (GLUT1 and GLUT5) fuels glycolysis, leading to increased production of glycolytic intermediates, including phosphoenolpyruvate (PEP) and pyruvate. Hexokinase (HK) and PKM2 act as key regulatory enzymes linking glycolysis to inflammatory signaling. Glycolytic reprogramming promotes stabilization of HIF-1α and activation of NF-κB and MAPK pathways, thereby enhancing transcription of proinflammatory mediators such as pro-IL-1β and NLRP3. Pharmacological inhibition of glycolysis by 2DG and accumulation of 2DG-6-phosphate attenuate inflammasome signaling. Mitochondrial metabolism further modulates NLRP3 activity through the TCA cycle. Accumulation of metabolites including succinate and fumarate, as well as altered succinate dehydrogenase (SDH) activity, promotes mtROS generation and inflammasome activation. By contrast, anti-inflammatory metabolites such as itaconate and BHB, suppress NLRP3 activation by limiting oxidative stress, inhibiting glycolytic flux or blocking K⁺ efflux. In addition, hexokinase, when dissociated from the mitochondrial outer membrane, senses NAC derived from peptidoglycan and activates the NLRP3 inflammasome. Fatty acids, including palmitate, and acetyl-CoA–dependent metabolic signaling influence inflammasome activity through effects on mitochondrial function and PTM pathways. The interaction between HK2 and ITPR3 attenuates Ca²⁺ release from the ER. This Ca²⁺ signaling modulates MAPK and STAT1 pathways, leading to the NLRP3 inflammasome inhibition.

Immunometabolic remodeling is closely associated with NLRP3 inflammasome activation (Fig. 3). Interestingly, the glycolytic enzyme hexokinase, dissociated from the mitochondrial outer membrane, acts as a pattern-recognition receptor that detects the *N*-acetylglucosamine (NAC) of peptidoglycan and activates the NLRP3 inflammasome. Notably, NAC-mediated NLRP3 inflammasome activation depends on recognition by hexokinase but not potassium efflux. Disruption of the glycolytic pathway using 2-deoxy-D-glucose (2DG) reduces NLRP3 inflammasome activation^53^. In addition, pyruvate kinase isoenzyme M2 (PKM2), an enzyme involved in aerobic glycolysis, participates in NLRP3 or absent in melanoma 2 (AIM2) inflammasome activation through EIF2AK2 phosphorylation^54^. Furthermore, the intermediate metabolite succinate stabilizes hypoxia-inducible factor-1α (HIF-1α), enhancing IL-1β production^55^. Conversely, itaconate, a metabolite produced by the enzyme ACOD1 encoded by *Acod1*/*irg1*, inhibits succinate dehydrogenase-mediated succinate oxidation, thereby exerting anti-inflammatory effects^56^. Moreover, fumarate suppresses nigericin-mediated pyroptosis by modifying gasdermin D cysteine residues into *S*-(2-succinyl)-cysteine, preventing gasdermin D interaction with caspases. These mechanisms explain the therapeutic action of fumarate in multiple sclerosis^57^. Collectively, these findings suggest that targeting specific metabolites or metabolic pathways could offer therapeutic strategies to regulate inflammasome activation and pyroptosis.

The saturated fatty acid palmitate is a well-established NLRP3 activator that induces endothelial dysfunction via NLRP3 inflammasome activation^58,59^. Fatty acid oxidation generates ketone bodies, such as β-hydroxybutyrate (BHB), which inhibit the NLRP3 inflammasome by suppressing K^+^ efflux^60^. In addition, fructose acts as a signaling molecule, strengthening the interaction between hexokinase 2 (HK2) and inositol 1,4,5-trisphosphate receptor type 3 (ITPR3), thereby reducing Ca^2+^ release from the ER. This decreased Ca^2+^ signaling impacts multiple intracellular pathways, including mitogen-activated protein kinase (MAPK) and signal transducer and activator of transcription 1 (STAT1), ultimately inhibiting NLRP3 inflammasome activation^61^. Further exploration of the crosstalk between immunometabolism and inflammasome signaling may enable the development of therapeutics for a range of diseases, such as metabolic disorders, atherosclerosis and inflammatory bowel disease, whose pathogenesis is potentially linked to metabolic and inflammasome dysregulation.

### Importance of Spatiotemporal Dynamics

An early study suggests that mitochondria-associated membranes play a important role in NLRP3–ASC contact formation, facilitating NLRP3 assembly. The acetylation-mediated transport of mitochondria via α-tubulin is critical for mitochondrial ASC apposition to NLRP3 in the endoplasmic reticulum (ER), which activates the NLRP3 inflammasome^62^. Recent studies indicate that the dynein adapter histone deacetylase 6 (HDAC6) is essential for microtubule transport and inflammasome assembly at the microtubule-organizing center (MTOC)^63^. MTOC is pivotal in NEK7 and NLRP3 interaction, enabling inflammasome activation and concurrent autophagy^2,63^. Polo-like kinase, linked to mitotic processes, enhances NLRP3 inflammasome activation by organizing MTOC structures^64^. Moreover, the cholesterol-regulating transcription factor SREBP2–SCAP complex associates with NLRP3, forming a ternary complex translocated from the ER to the Golgi apparatus for optimal inflammasome assembly^65^.

Emerging evidence highlights that electrostatic interactions between the polybasic region of NLRP3 and negatively charged lipids are vital for recruiting NLRP3 to the TGN, connecting these events to MTOC. The interaction of NLRP3 with phosphatidylinositol-4-phosphate (PtdIns4P) on the dTGN is critical for NLRP3 aggregation, ASC polymerization and inflammasome activation in response to diverse stimuli^66^. Additional studies have identified two specific NLRP3 palmitoylation sites facilitated by the enzyme zDHHC1, which mediates NLRP3 translocation among mitochondria, TGN and endosomes. Ultimately, NLRP3 gathers at MTOC, where LATS1 and LATS2 phosphorylate NLRP3 to interact with NEK7, fully activating the inflammasome^67^. A cryo-electron microscopy study revealed that NLRP3 forms an open octamer disrupted by NEK7 into NEK7/NLRP3 monomers/dimers, preceding disk-like inflammasome assembly^68^.

IKKβ activation in induced pluripotent stem cell-derived human macrophages during priming is critical for NLRP3 recruitment to phosphatidylinositol-4-phosphate (PI4P) on the trans-Golgi network, where tethering to PI4P activates the NLRP3 inflammasome^69^. This process appears NEK7 independent, suggesting that IKKβ-mediated pathways play a major role in NLRP3 priming and inflammasome activation in human myeloid cells^69^. However, it is unclear which molecular target is phosphorylated by IKKβ or if NEK7-independent IKKβ activation is vital in vivo for NLRP3 priming and activation. Recently, S-acylation of NLRP3 at Cys-130 was found to facilitate its recruitment to the Golgi for activation^70^. These findings provide valuable insights into the spatiotemporal regulation and activation of the NLRP3 inflammasome, but further research is required to elucidate the precise sequential events regulating inflammasome activation, including PTM of each component, scaffold interactions and pro-caspase-1 cleavage under specific contexts.

## PTMs regulating NLRP3 inflammasome activation

PTMs of NLRP3 play a crucial role in its regulation and function, particularly in the context of inflammation and innate immunity. NLRP3 is a vital component of the inflammasome, a multiprotein complex that impacts the host immune response by initiating inflammatory processes. The key PTMs regulating NLRP3 are as follows. In addition, we show in this Review how the major PTMs of NLRP3 can regulate inflammasome activation (Table 1, main table).

**Table 1:** The post-translational modification of NLRP3.

Ubiquitination/deubiquitination					
Enzyme	Modification site	Regulation	Potential mechanisms	Ubiquitin chain types	Ref.
Ubc13	Lys565/Lys687	Activating	Promotes NLRP3 K63-linked polyubiquitination.	K63	^71^
RNF125	LRR	Inhibitory	Initiates K63-linked ubiquitination of the NLRP3 LRR domain	K63	^72^
Cbl-b	Lys496 (NACHT)	Inhibitory	Targets NLRP3 at K496 for K48-linked ubiquitination and proteasome-mediated degradation	K48	^72^
TRIM31		Inhibitory	Promoting NLRP3 polyubiquitination	K48	^73^
USP13	K192/K496	Inhibitory	Inhibiting proteasomal degradation of NLRP3.		^74^
MARCH7	LRR	Inhibitory	Facilitating NLRP3 ubiquitination and degradation	K48	^75^
Cullin1	Lys689	Inhibitory	Facilitating K63-linked ubiquitination	K63	^76^
FBXL2	Lys689	Inhibitory	Mediating NLRP3 ubiquitination to inhibit inflammasome activation		^77^
ARIH2	Lys48/lys63	Inhibitory	Promotes NLRP3 polyubiquitination.	K48/ K63	^78^
gp78		Inhibitory	Inhibiting the oligomerization and subcellular translocation of NLRP3		^79^
PARK2		Inhibitory	Promoting NLRP3 ubiquitination		^80^
MARCH5	K324/ K430	Activating	Promotes NLRP3–NEK7 complex formation and inflammasome activation	K27	^81^
TRIM65		Inhibitory	Promotes lys48- and lys63- linked ubiquitination of NLRP3	K48/ K63	^82^
HUWE1	Lys21/22/24	Activating	Led to inflammasome assembly, ASC speck formation, and sustained caspase-1 activation	K27	^83^
YAP	Lys380	Inhibitory	Inhibits the E3 activity of β-TrCPt1 leading the proteasomal degradation of NLRP3	K27	^84^
Pellino2		Activating	Enhances NLRP3 ubiquitination	K63	^85^
IRAK1		Inhibitory	Acts to suppress NLRP3 ubiquitination		^85^
TRIM24		Inhibitory	Facilitates the ubiquitination of NLRP3		^86^
TRIM40		Inhibitory	Mediates the ubiquitination of NLRP3 through the RING domain		^87^
TRIM50		Inhibitory	Suppression of NLRP3 ubiquitination		^88^
USP1		Activating	Removes the K48-linked polyubiquitination of NLRP3	K48	^89^
UAF1		Activating	Removes the K48-linked polyubiquitination of NLRP3	K48	^89^
USP30		Activating	Inhibition of maintaining ubiquitination of NLRP3		^90^
USP50		Activating	Stabilizing NLRP3 and preventing its proteasomal degradation	K48	^91^
USP9X		Activating	Induces deubiquitination of NLRP3		^92^
USP25	K243	Activating	Disrupting NLRP3–ASC interactions and ASC oligomerization.	K63	^93^
STING		Activating	Localizing NLRP3 promotes inflammasome formation and attenuates polyubiquitination	K48/ K63	^94,95^
BRCA2/ABRO1		Activating	Deubiquitinates the LRR domain of NLRP3 during inflammasome activation		^96–98^
YOD1		Inhibitory	Removal of K33-linked ubiquitination of NLRP3	K33	^99^
USP7		Activating	Promotes inflammasome formation		^100^
USP47		Activating	Promotes inflammasome formation		^100^
Vitamin D receptor		Inhibitory	Blocking BRCC3-mediated deubiquitination		^101^
STAMBP		Inhibitory	Increased NLRP3 K63 chain polyubiquitination	K63	^102^

Phophorylation/dephosphorylation				
Enzyme	Modification site	Regulation	Potential mechanisms	Ref.
PKD	Ser295(h)/Ser293(m)	Activating	To release NLRP3 from mitochondria	^103^
PKA	Ser295	Inhibitory	Impairing ATPase activity	^104,105^
AKT	Ser5(h)/Ser3(m)	Inhibitory	Impairs the interaction between DDX3X and NLRP3	^106^
MINK1	Ser725(h)/Ser728(m)	Activating	Direct interaction between MINK1 and the NLRP3	^107^
Pyk2	Tyr146	Activating	Phosphorylates Tyr146 of ASC	^108^
BRCC3		Inhibitory	Induction of NLRP3 deubiquitination	^109^
PGK1	S448/S449		Phosphorylates NLRP3 at S448/S449 and promotes inflammasome activation	^110^
Lyn	Tyr918	Inhibitory	Promotes the ubiquitination and proteasomal degradation of NLRP3	^111^
BTK	Y136/Y140/Y143/Y168	Activating	To shift NLRP3 localization from intact to dispersed Golgi membranes	^112^
EphA2	Tyr132(h)/Tyr136(m)	Inhibitory	Induces its phosphorylation at Tyr132 of NLRP3	^113^
PTPN22	Tyr858(h)/Tyr861(m)	Inhibitory	Dephosphorylates NLRP3 at tyrosine residue Tyr861	^114,115^
PP2A	Ser5(h)/Ser3(m)	Activating	Dephosphorylation at S5 of NLRP3	^116^
TBK1	Ser5(h)/Ser3(m)	Inhibitory	Regulating AKT and counteracting PP2A	^117^
IKKε	Ser5(h)/Ser3(m)	Inhibitory	Regulating AKT and counteracting PP2A	^117^

Acetylation/deacetylation				
Enzyme	Modification site	Regulation	Potential mechanisms	Ref.
KAT5	Lys24	Activating	Catalyzes the acetylation of lysine 24 on NLRP3	^118^
SIRT2		Inhibitory	Deacetylation of NLRP3	^119^
Tau	K21/K22/K24	Activating	Promoting inflammasome activation	^120^
HDAC2		Inhibitory	Suppresses NLRP3 transcription	^121^
HDAC6		Inhibitory	Promoting NLRP3-dependent pyroptosis	^122^
HDAC10	K496	Inhibitory	Suppressing NLRP3 inflammasome activation	^123^

SUMOylation				
Enzyme	Modification site	Regulation	Potential mechanisms	Ref.
Ubc9	Lys204	Activating	Promotes ASC oligomerization and inflammasome assembly	^124^
SENP3	Lys204	Inhibitory	Reducing ASC speck formation and inflammasome activation	^125^
Ox-LDL		Inhibitory	Decreases CD36 expression and cholesterol absorption	^126^
MAPL(MUL1)	Lys689	Activating	Enhanced caspase-1 activation and IL-1β release	^125^
SENP6	Lys689	Inhibitory	Promotes NLRP3-dependent ASC oligomerization and caspase-1 activation	^125^
SENP7	Lys689	Inhibitory	Promotes NLRP3-dependent ASC oligomerization and caspase-1 activation	^125^
Zmiz1		Activating	Increasing NLRP3 SUMOylation	^127^
TRIM28		Activating	Enhancer of NLRP3 inflammasome activation	^128^

Alkylation				
Compounds	Modification site	Regulation	Potential mechanisms	Ref.
BT4-ONE		Inhibitory	Hindering the assembly of the NLRP3 inflammasome	^129^
Parthenolide		Inhibitory	Inhibited the ATPase activity of NLRP3	^130^
Bay 11-7082		Inhibitory	Inhibited the ATPase activity of NLRP3	^131^
MNS		Inhibitory	Prevented NLRP3-mediated ASC speckle formation and oligomerization by NLRP3 agonists	^132^

Palmitoylation				
Enzyme	Modification site	Regulation	Potential mechanisms	Ref.
ABHD8		Inhibitory	Reducing NLRP3 inflammasome activation	^133^
zDHHC12	Cys844	Inhibitory	Promotes chaperone-mediated degradation of NLRP3	^133^
zDHHC5	Cys837/Cys838	Activating	Enhances the inflammasome assembly	^134^
zDHHC1		Activating	Facilitates movement across cell membranes	^67^
ZDHHC17	Cys419	Activating	Promotes NIMA-related kinase 7 (NEK7)–NLRP3 interaction	^135^
ZDHHC7		Activating	Promoting NLRP3 activation	^136^

ADP ribosylation				
Enzyme	Modification site	Regulation	Potential mechanisms	Ref.
PARP-1		Activating	Binds to and PARylates NLRP3	^137^
Mycoplasma pneumoniae		Activating	Catalyzing the ADP ribosylation of NLRP3	^138^

ABHD8, α/β-hydrolase domain-containing 8; ARIH2, Ariadne homolog 2; BRCA2/ABRO1, BRCA2-containing complex subunit 3/Abraxas brother protein 1; BRCC3, BRCA1/BRCA2-containing complex subunit 3; Cbl-b,Cbl proto-oncogene B; EphA2, EPH receptor A2; FBXL2, F-box/LRR-repeat protein 2; HDAC, histone deacetylase 2; HUWE1, HECT, UBA and WWE domain-containing E3 ligase 1; IRAK1, interleukin 1 receptor associated kinase 1; KAT5, Lysine acetyltransferase 5; MARCH, membrane associated ring-CH-type finger; MINK1, (Msn).NIK-related kinase 1; PARK2, Parkin RBR E3 ubiquitin protein ligase 2; Pyk2, proline-rich tyrosine 2; RNF125, Ring Finger Protein 125; SENP6, Sentrin/SUMO-specific protease 6; SENP7, Sentrin/SUMO-specific protease 7; SIRT2, sirtuin 2; STAMBP, STAM binding protein; STING, stimulator of interferon genes; TBK1, TANK binding kinase 1; TRIM28, E3 SUMO ligase tripartite motif-containing protein 28; TRIM, tripartite motif; USP, ubiquitin-specific peptidase; YAP, Yes-associated protein; zDHHC, Zinc finger DHHC (Asp-His-His-Cys)-type-containing.

### Ubiquitination

Extensive studies have reported the ubiquitination of NLRP3 for its degradation and suppression of inflammasome complex assembly. The ubiquitination of NLRP3 by various E3 ligases highlights their roles in suppressing inflammasome activation. For example, Ubc13 associates with NLRP3 and promotes its K63-linked polyubiquitination, with lys565 and lys687 identified as K63-linked sites through mass spectrometry and biochemical analysis^71^. Similarly, E3 ubiquitin ligases RNF125 and Cbl-b regulate NLRP3 inflammasome activity via K63- and K48-linked ubiquitination, respectively, leading to proteasome-mediated degradation of NLRP3^72^. In addition, TRIM31 binds NLRP3, promoting its polyubiquitination and proteasomal degradation^73^. Moreover, USP13 interacts with NLRP3 by competing with the E3 ubiquitin ligase TRIM31, preventing TRIM31-mediated ubiquitination of NLRP3 at K192 and K496, thereby inhibiting proteasomal degradation of NLRP3. USP13 deficiency reduces NLRP3 protein expression in both human and mouse macrophages, inhibiting the assembly and activation of the NLRP3 inflammasome^74^. MARCH7 facilitates dopamine-mediated inhibition of the NLRP3 inflammasome by promoting its ubiquitination and degradation^75^. Cullin1, part of the Skp1–Cullin1–F-box complex, interacts with NLRP3, facilitating K63-linked ubiquitination at lys689, effectively inhibiting NLRP3 activation without degradation^76^. FBXL2 recognizes Trp-73 and targets lys689 in NLRP3 for ubiquitin ligation, promoting proteasomal degradation and inhibiting inflammasome activation^77^. Ariadne homolog 2 (ARIH2) ubiquitinates NLRP3 at lys48 and lys63, negatively regulating NLRP3 priming in macrophages^78^. The membrane-bound E3 ubiquitin ligase gp78 mediates mixed ubiquitination of NLRP3, inhibiting its oligomerization and subcellular translocation, thereby suppressing inflammasome activation^79^. Last, PARK2 attenuates house dust mite-induced inflammatory responses, proptosis, and barrier dysfunction in BEAS-2B cells by interacting with and ubiquitinating NLRP3^80^.

Several other E3 ligases have been implicated in regulating NLRP3 inflammasome activation through NLRP3 ubiquitination. The mitochondria-associated E3 ligase MARCH5 promotes NLRP3–NEK7 complex formation and inflammasome activation by inducing K27-linked polyubiquitination at the K324 and K430 residues of NLRP3^81^. Inhibition or deletion of TRIM65 notably enhances NLRP3 inflammasome activation in THP-1 cells and bone marrow-derived macrophages (BMDMs)^82^. HUWE1, an E3 ubiquitin ligase, interacts with NLRP3 and NLRC4 via the HIN domain of AIM2 and the NACHT domains of NLRP3 and NLRC4, mediating inflammasome activation as part of the host defense against bacterial infection^83^. The transcription coactivator YAP physically interacts with NLRP3, maintaining its stability by suppressing interaction with the E3 ligase β-TrCPt1. YAP inhibits β-TrCPt1’s E3 activity, preventing proteasomal degradation of NLRP3 via K27-linked ubiquitination at lys380^84^. Another E3 ligase, Pellino2, enhances NLRP3 ubiquitination during the priming phase (signal 1), promoting inflammasome activation, while IRAK1 has the opposite effect^85^.

Members of the tripartite motif (TRIM) family also regulate NLRP3 inflammasome activity via ubiquitination modulation. For instance, TRIM24 negatively regulates NLRP3/caspase-1/IL-1β-mediated proptosis and human embryonic stem cell migration. TRIM24 interacts with NLRP3, facilitating its ubiquitination in ectopic human embryonic stem cells^86^. Similarly, TRIM40 mediates NLRP3 ubiquitination through its RING domain, inhibiting inflammasome activation and glomerular mesangial cell proliferation caused by IgA1^87^. By contrast, TRIM50 interacts with NLRP3 and suppresses its ubiquitination, promoting NLRP3 inflammasome activation^88^.

### Deubiquitination

Most studies on the deubiquitination of NLRP3 by deubiquitination enzymes (DUBs) and related factors highlight their role in facilitating NLRP3 inflammasome activation and associated pathologies. The DUB complex, comprising ubiquitin-specific processing protease 1 (USP1) and USP1-associated factor-1 (UAF1), removes K48-linked polyubiquitination from NLRP3, stabilizing its protein expression and promoting inflammasome activation^89^. Similarly, USP30 activates the NLRP3 inflammasome by deubiquitinating NLRP3^90^, its knockdown or inhibition via MF-094 suppresses inflammasome activation by maintaining NLRP3 ubiquitination. In hepatocellular carcinoma, ubiquitin-specific protease 50 (USP50) directly interacts with NLRP3 and functions as a deubiquitinase that selectively removes K48-linked polyubiquitin chains, thereby stabilizing NLRP3 and preventing its proteasomal degradation^91^. USP9X enhances NLRP3 protein levels by inducing its deubiquitinating, thereby activating the inflammasome without altering mRNA expression^92^. USP25 directly binds to NLRP3 and removes K63-linked ubiquitin chains from residue K243 through its catalytic site C178, thereby disrupting NLRP3–ASC interactions and ASC oligomerization. This ultimately suppresses NLRP3 activation and pyroptosis in cardiomyocytes^93^.

In cytosolic DNA stimulation and herpes simplex virus type 1 infection, the stimulator of interferon genes (STING) acts as a DUB, enhancing NLRP3 inflammasome activation by interacting with NLRP3 and reducing its K48- and K63-linked polyubiquitination^94^. Notably, oxidative stress conditions also induce cGAS–STING activation and NLRP3-mediated inflammasome activation^95^. Priming signals from TLR4 or TLR2 stimulation induce the expression of Abraxas brother protein 1 (ABRO1), a subunit of the BRCA2-containing complex subunit 3 (BRCC3, also known as BRCC36). This complex deubiquitinates the LRR domain of NLRP3 during inflammasome activation^96,97^. Cholesterol-induced inflammasome activation in Tet2-deficient macrophages is further enhanced through JNK1- and BRCC3-mediated NLRP3 deubiquitination^98^.

Other DUBs are involved in suppressing NLRP3 inflammasome activity. For instance, the deubiquitinase YOD1 interacts with NLRP3 and removes K33-linked ubiquitination, suppressing NLRP3 expression and activation, which mitigates organ injury during methicillin-resistant *Staphylococcus aureus* sepsis^99^. Ubiquitin-specific peptidases USP7 and USP47 are critical for removing ubiquitin from NLRP3 and inhibiting ASC speck formation, with both exhibiting functional redundancy in deubiquitinating NLRP3^100^. In addition, the vitamin D receptor inhibits NLRP3 activation by blocking BRCC3-mediated deubiquitination^101^. The deubiquitinase STAM binding protein regulates NLRP3-mediated inflammatory responses; its downregulation via CRISPR–Cas9 increases cytokine secretion and inflammasome activity, highlighting its role in limiting excessive activation^102^.

Given that NLRP3 inflammasome activation is tightly regulated by ubiquitination and deubiquitination, understanding how DUBs modulate NLRP3 and identifying associated signaling pathways could pave the way for novel therapeutic strategies targeting NLRP3-related pathologies.

### Phosphorylation and dephosphorylation

During priming, NLRP3 undergoes phosphorylation at Ser295 in humans (Ser293 in mice) by protein kinase D (PKD), an effector of diacylglycerol, at the Golgi apparatus near mitochondria-associated ER membranes, where NLRP3 and ASC converge to assemble the inflammasome complex^103^. Conversely, phosphorylation of Ser295 by protein kinase A (PKA) inhibits NLRP3 by reducing the ATPase activity of its NACHT domain, a step crucial for oligomerization^104^. In response to bile acids or prostaglandin E2 signaling, PKA-mediated phosphorylation of S295 diminishes NLRP3 ATPase activity, thereby impairing inflammasome formation. Notably, this phosphorylation is impaired in CAPS-mutant NLRP3^105^. AKT-induced phosphorylation of DDX3X, a novel NLRP3 inflammasome component, disrupts the interaction between DDX3X and NLRP3, affecting inflammasome activity^106^. In addition, phosphorylation of Ser725 by misshapen (Msn)/NIK-related kinase 1 (MINK1) is essential for NLRP3 inflammasome priming. MINK1 directly interacts with the NLRP3 LRR domain, and its deficiency reduces NLRP3 activation, mitigating inflammatory responses in sepsis and peritonitis models^107^. PyK2 phosphorylates Tyr146 of ASC, enhancing IL-1β secretion^108^. NLRP3–NEK7 binding facilitates deubiquitination by BRCC3 and inflammasome assembly. Phosphomimetic mutants of NLRP3 exhibit increased ubiquitination and degradation compared to wild type^109^. Phosphoglycerate kinase 1 (PGK1), a key glycolytic enzyme, also functions as a kinase that directly regulates NLRP3 inflammasome activation following lipopolysaccharide stimulation. PGK1 phosphorylates NLRP3 at S448/S449, which in turn recruits USP14 to facilitate NLRP3 deubiquitination and promote inflammasome activation^110^.

Src family kinase Lyn phosphorylates Tyr918, promoting NLRP3 ubiquitination and proteasomal degradation^111^. Similarly, Bruton’s tyrosine kinase (BTK) phosphorylates NLRP3 at Y136, Y140, Y143 and Y168 within the PYD–NACHT polybasic linker, altering its charge from net positive to net negative. This shift redistributes NLRP3 from intact to dispersed Golgi membranes enhancing ASC interaction and IL-1β release. BTK inhibition blocks NLRP3 inflammasome formation and IL-1β secretion, offering a therapeutic target^112^. The transmembrane tyrosine kinase EphA2 directly interacts with NLRP3 and induces Tyr132 phosphorylation^113^. Protein tyrosine phosphatase nonreceptor 22 (PTPN22) dephosphorylates Tyr861 of NLRP3, activating the inflammasome and promoting IL-1 secretion^114,115^.

In the resting state, unintended NLRP3 oligomerization is prevented by AKT-induced phosphorylation of S5, which creates electrostatic repulsion, hindering interactions with the PYD of NLRP3. Dephosphorylation of S5 by phosphatase 2A (PP2A) primes NLRP3 for downstream signaling, but additional stimuli are needed for full inflammasome assembly^116^. TBK1 and IKKε were recently identified as regulators that cooperate in controlling AKT and counteracting PP2A to limit NLRP3 activity. These kinases also target sites other than S5, indicating broader effects beyond AKT regulation^117^.

### Acetylation/deacetylation

In response to the second signal for NLRP3 inflammasome activation, KAT5, a member of the MYST histone acetyltransferase family, catalyzes lys24 acetylation on murine NLRP3. This modification promotes NLRP3 oligomerization and enhances its binding to ASC and NEK7^118^. Acetylation is essential for NLRP3 self-aggregation and full inflammasome activation. SIRT2-mediated deacetylation of NLRP3 reduces inflammasome activation, mitigating aging-associated inflammation and insulin resistance^119^. Tau acetylates NLRP3 at K21, K22 and K24 within its PYD domain, thereby promoting inflammasome activation^120^. In colorectal cancer cells, histone deacetylase 2 (HDAC2) suppresses NLRP3 transcription by preventing H3K27 acetylation-dependent recruitment of the BRD4/p-p65 complex^121^. In aged mice, splenectomy performed under sevoflurane anesthesia increases HDAC6 expression, which contributes to postoperative cognitive dysfunction by promoting NLRP3-dependent pyroptosis through the HSP90/HSP70 pathway in hippocampal microglia^122^. In addition, HDAC10 directly interacts with NLRP3 and induces deacetylation at the K496 residue, thereby suppressing NLRP3 inflammasome activation and mitigating the resulting acute inflammatory damage^123^.

### SUMOylation

The small ubiquitin-like modifier (SUMO) protein is evolutionarily conserved and ubiquitously expressed in eukaryotes. As a member of the ubiquitin-like family, it modifies proteins via PTMs. SUMOylation of NLRP3 at Lys204 promotes activation, while SUMOylation at Lys689 suppresses it. SUMO1 induces Ubc9-mediated SUMOylation at Lys204, facilitating ASC oligomerization and inflammasome assembly^124^. SUMO-specific protease 3 (SENP3) mediates SUMOylation, reducing ASC speck formation and inflammasome activation^125^. Ox-LDL-stimulated macrophages show reduced NLRP3 SUMOylation, which decreases CD36 expression and cholesterol absorption^126^. Mitochondrial-anchored protein ligase (MAPL), also known as mitochondrial E3 ubiquitin protein ligase 1 (MUL1), mediates RING finger-dependent SUMOylation of NLRP3 at Lys689, enhancing caspase-1 maturation and IL-1 release in murine macrophages and human cells^125^. SENP6 and SENP7 deSUMOylate NLRP3 at Lys689, promoting NLRP3-dependent ASC oligomerization and caspase-1 activation. MAPL does not influence AIM2 inflammasome activation^125^. Zinc finger MIZ-type-containing 1 (Zmiz1) has been reported to enhance SUMOylation of the NLRP3 inflammasome. In vitro, Zmiz1 overexpression markedly increased NLRP3 SUMOylation, thereby promoting SGC activation and inflammation while inhibiting neuronal autophagy^127^. SUMOylation can also regulate protein function by affecting ubiquitination. The E3 SUMO ligase TRIM28 catalyzes SUMO1/2/3-conjugated SUMOylation of NLRP3, inhibiting its ubiquitination and proteasomal degradation, thereby enhancing inflammasome activation^128^.

### Alkylation

The alkylation of NLRP3 impacts inflammasome activation by altering its ATP affinity. Mutations in the NACHT domain of NLRP3 impair ATPase activity, reducing ATP binding and subsequently decreasing caspase-1 activation, IL-1β production, apoptosis, macromolecular complex formation, self-association and interaction with ASC^129^. In addition, NLRP3 alkylation is linked to its ubiquitination. Alkylation by BT4-ONE impairs ATPase activity, hindering inflammasome assembly and increasing NLRP3 ubiquitination^130^. In vitro studies revealed that parthenolide and Bay 11-7082 inhibit NLRP3 ATPase activity^131^. The NLRP3 inhibitor 3,4-methylenedioxy-β-nitrostyrene (MNS) specifically blocks NLRP3-mediated ASC speck formation and oligomerization induced by NLRP3 agonists^132^.

### Palmitoylation

ABHD8, a member of the α/β-hydrolase domain-containing (ABHD) family, functions as a scaffold that recruits the palmitoyltransferase ZDHHC12 to NLRP3, promoting its palmitoylation and subsequent chaperone-mediated autophagy-mediated degradation. Consequently, ABHD8 deficiency stabilizes the NLRP3 protein and enhances NLRP3 inflammasome activation^133^. ZDHHC5 facilitates palmitoylation of NLRP3 at the LRR domain, enhancing inflammasome assembly and activation, while ABHD17A depalmitoylates NLRP3^134^. ZDHHC1 mediates palmitoylation to enable NLRP3 trafficking between membranes such as mitochondria, Golgi and endosomes, promoting inflammation during the priming phase and leading to full activation^67^. ZDHHC17 catalyzes palmitoylation at Cys419, enhancing the NIMA-related kinase 7 (NEK7)–NLRP3 interaction and inflammasome activation^135^. Palmitoyltransferase ZDHHC7 mediates NLRP3 palmitoylation in response to mechanical cues, thereby promoting NLRP3 activation^136^.

### ADP ribosylation

Poly ADP-ribose polymerase-1 (PARP-1), part of the ADP-ribosyl transferase family, translocates to the cytosol upon ATP stimulation, binding and PARylating NLRP3 to promote inflammasome assembly^137^. The vacuolating toxin from *Mycoplasma pneumoniae*, known as the community-acquired respiratory distress syndrome toxin, directly interacts with NLRP3, triggering its ADP ribosylation^138^.

Understanding PTMs is essential for developing therapeutic strategies to regulate NLRP3 activity in inflammatory diseases. Targeting PTMs offers a way to control inappropriate NLRP3 activation and mitigate inflammatory responses. PTMs are critical regulators of the NLRP3 inflammasome activation cycle; however, further research is required to elucidate how different PTMs interact and collectively influence NLRP3 activation and inflammasome assembly. Targeting enzymes responsible for these modifications presents a promising therapeutic avenue for managing proinflammatory diseases linked to aberrant NLRP3 activation.

## NLRP3-interacting partners with unknown mechanisms

This section explores molecules that interact with components of the NLRP3 inflammasome complex to activate or inhibit its function, though their regulatory mechanisms may not directly involve PTMs. We also summarize them in Table 2.

**Table 2:** NLRP3-interaction regulator.

protein	Interacting site	Regulation	Potential mechanisms	Ref.
STAT3	PYD/LRR domain	Activating	Translocate NLRP3 to mitochondria	^139^
UCH-L1	NACHT domain	Activating	Induced IL-1β production	^140^
SERTAD1	NACHT domain	Activating	Induced NLRP3 ubiquitination	^141^
A20		Inhibitory	Disrupts the interacting between MEK7 and NLRP3	^142^
HAX-1		Inhibitory	Competitive binding of NLRP3–ASC	^143^
MARCH2		Inhibitory	Interacts with PGAM5 to degradation of NLRP3	^144^
ZNFX1		Inhibitory	Retains NLRP3 in the cytoplasm	^145^
ABHD8		Inhibitory	Promotes NLRPs degradation	^146^
HECTD3		Inhibitory	Blocks the interaction between NEK7 and NLRP3	^147^

### Positive regulators of the NLRP3 inflammasome

Extensive studies have examined molecules interacting with NLRP3 and their role in upregulating its function, often in the context of disease pathologies. For example, signal transducer and activator of transcription-3 (STAT3), a protein highly implicated in inflammation and cancer, interacts with NLRP3 and phosphorylates it at Ser727. This interaction enables STAT3-bound NLRP3 translocation to mitochondria, promoting inflammasome activation^139^. Similarly, ubiquitin C-terminal hydrolase 1 (UCH-L1) interacts with the NLRP3 NACHT domain, leading to inflammasome activation, particularly in microglial cells^140^. Moreover, SERTA domain-containing protein (SERTAD1), an adapter protein that binds NLRP3 and reduces Cullin1-mediated K63-linked polyubiquitination, initiates inflammasome activation^141^. Notably, SERTAD1 mRNA is upregulated in patients with autoimmune disease^141^. Modulating these interacting partners of NLRP3 may provide pharmacological tools to control inflammasome hyperactivation, potentially addressing a range of inflammation-associated pathologies^64^.

### Negative regulators of the NLRP3 inflammasome

A20, a well-characterized deubiquitinase, plays a negative regulatory role in NLRP3 inflammasome activity by binding to NEK7 and mediating its K48-linked ubiquitination, facilitating proteasomal degradation. A20 also disrupts the NEK7–NLRP3 interaction within the inflammasome assembly^142^. In cerebral ischemia–reperfusion injury, the prosurvival protein hematopoietic cell-specific protein-associated protein X1 (HAX-1) inhibits NLRP3 inflammasome activation and microglial pyroptosis by competitively binding to NLRP3 and preventing its interaction with ASC^143^. Similarly, the E3 ubiquitin ligase membrane-associated RING finger protein 2 (MARCH2) negatively regulates NLRP3 inflammasome activity and cardiomyocyte pyroptosis by interacting with phosphoglycerate mutase 5 (PGAM5) to promote its K48-linked polyubiquitination and degradation, suppressing PGAM5–MAVS condensation^144^.

Recent findings have also highlighted the negative regulatory role of zinc finger NFX1-type-containing 1 (ZNFX1), a zinc finger protein, in NLRP3 inflammasome activation. ZNFX1 interacts with NLRP3 in the cytoplasm, independent of the TGN38^+^/TGN46^+^ vesicles associated with the TGN^145^. Moreover, ABHD8, a member of the ABHD family, promotes NLRP3 degradation by forming a scaffold with ZDHHC12, facilitating NLRP3 palmitoylation through chaperone-mediated autophagy^146^. The E3 ubiquitin ligase HECT domain E3 ubiquitin protein ligase 3 (HECTD3) also interacts with the NACHT/LRR domains of NLRP3, blocking the NLRP3–NEK7 interaction and preventing NLRP3 oligomerization, independent of its E3 ligase activity^147^.

## Conclusions

Over the past few decades, substantial progress has been made in understanding NLRP3 inflammasome activation. While advancements have shed light on the complex molecular events involved, several critical areas remain unexplored. Specifically, there are at least three key gaps in our current knowledge and the potential impact of addressing them.

First, the mechanisms by which various signals and stimuli trigger NLRP3 inflammasome assembly across different cell types remain unclear. It is an open question whether diverse exogenous and endogenous signals converge on a single upstream pathway or if distinct mechanisms are employed for each type of stimulus. Second, while recent structural studies have provided snapshots of the NLRP3 inflammasome complex, the dynamic, real-time steps of its assembly, particularly how components and interacting partners come together across various intracellular organelles, are not fully understood. Investigating these organelle-specific interactions and crosstalk could reveal new therapeutic targets, enabling researchers to modulate inflammasome assembly at precise locations under pathological conditions. Tracking the spatiotemporal dynamics of inflammasome activation may also aid in developing more selective drug candidates that target specific stages of the assembly process. Third, substantial progress has been made in identifying key PTMs that regulate NLRP3 inflammasome activation. However, a comprehensive understanding of how these modifications interact with or influence one another is still lacking. It is also unknown whether certain PTMs play a more prominent role in specific cell types or pathological conditions. Given the reversibility of many PTMs, they could offer novel strategies to precisely modulate inflammasome activity in a disease-specific manner. Future studies addressing these research gaps could enhance the integrated understanding of the explored mechanisms into a more comprehensive mechanistic framework.

A deeper understanding of the NLRP3 inflammasome, its regulatory mechanisms and the molecular events driving its activation could importantly enhance our ability to identify therapeutic intervention points. These advancements hold the promise of reducing the severity or preventing the progression of a wide range of NLRP3-related inflammatory diseases, ultimately improving patient outcomes.

## References

[1] Huang, Y., Xu, W. & Zhou, R. NLRP3 inflammasome activation and cell death. *Cell. Mol. Immunol.* **18**, 2114–2127 (2021). 34321623 10.1038/s41423-021-00740-6 PMC8429580

[2] Xu, J. & Nunez, G. The NLRP3 inflammasome: activation and regulation. *Trends Biochem. Sci.* **48**, 331–344 (2023). 36336552 10.1016/j.tibs.2022.10.002 PMC10023278

[3] Swanson, K. V., Deng, M. & Ting, J. P. The NLRP3 inflammasome: molecular activation and regulation to therapeutics. *Nat. Rev. Immunol.* **19**, 477–489 (2019). 31036962 10.1038/s41577-019-0165-0 PMC7807242

[4] Fu, J. & Wu, H. Structural mechanisms of NLRP3 inflammasome assembly and activation. *Annu. Rev. Immunol.* **41**, 301–316 (2023). 36750315 10.1146/annurev-immunol-081022-021207 PMC10159982

[5] O’Keefe, M. E., Dubyak, G. R. & Abbott, D. W. Post-translational control of NLRP3 inflammasome signaling. *J. Biol. Chem.* **300**, 107386 (2024). 38763335 10.1016/j.jbc.2024.107386 PMC11245928

[6] Sharma, B. R. & Kanneganti, T. D. NLRP3 inflammasome in cancer and metabolic diseases. *Nat. Immunol.* **22**, 550–559 (2021). 33707781 10.1038/s41590-021-00886-5 PMC8132572

[7] Chen, Y. et al. The NLRP3 inflammasome: contributions to inflammation-related diseases. *Cell. Mol. Biol. Lett.* **28**, 51 (2023). 37370025 10.1186/s11658-023-00462-9 PMC10303833

[8] Zhou, J. et al. Pyroptosis and degenerative diseases of the elderly. *Cell Death Dis.* **14**, 94 (2023). 36755014 10.1038/s41419-023-05634-1 PMC9908978

[9] Schroder, K. & Tschopp, J. The inflammasomes. *Cell* **140**, 821–832 (2010). 20303873 10.1016/j.cell.2010.01.040

[10] Sharif, H. et al. Structural mechanism for NEK7-licensed activation of NLRP3 inflammasome. *Nature* **570**, 338–343 (2019). 31189953 10.1038/s41586-019-1295-z PMC6774351

[11] Cai, X. et al. Prion-like polymerization underlies signal transduction in antiviral immune defense and inflammasome activation. *Cell* **156**, 1207–1222 (2014). 24630723 10.1016/j.cell.2014.01.063 PMC4034535

[12] Xiao, L., Magupalli, V. G. & Wu, H. Cryo-EM structures of the active NLRP3 inflammasome disc. *Nature* **613**, 595–600 (2023). 36442502 10.1038/s41586-022-05570-8 PMC10091861

[13] He, Y., Zeng, M. Y., Yang, D., Motro, B. & Nunez, G. NEK7 is an essential mediator of NLRP3 activation downstream of potassium efflux. *Nature* **530**, 354–357 (2016). 26814970 10.1038/nature16959 PMC4810788

[14] McDonald, B. et al. Intravascular danger signals guide neutrophils to sites of sterile inflammation. *Science* **330**, 362–366 (2010). 20947763 10.1126/science.1195491

[15] Mariathasan, S. et al. Cryopyrin activates the inflammasome in response to toxins and ATP. *Nature* **440**, 228–232 (2006). 16407890 10.1038/nature04515

[16] Wang, Y. et al. Cellular localization of NLRP3 inflammasome. *Protein Cell* **4**, 425–431 (2013). 23609011 10.1007/s13238-013-2113-2 PMC4875552

[17] Kanneganti, T. D. et al. Pannexin-1-mediated recognition of bacterial molecules activates the cryopyrin inflammasome independent of Toll-like receptor signaling. *Immunity* **26**, 433–443 (2007). 17433728 10.1016/j.immuni.2007.03.008

[18] Sutterwala, F. S. et al. Critical role for NALP3/CIAS1/Cryopyrin in innate and adaptive immunity through its regulation of caspase-1. *Immunity* **24**, 317–327 (2006). 16546100 10.1016/j.immuni.2006.02.004

[19] Hornung, V. et al. Silica crystals and aluminum salts activate the NALP3 inflammasome through phagosomal destabilization. *Nat. Immunol.* **9**, 847–856 (2008). 18604214 10.1038/ni.1631 PMC2834784

[20] Kuroda, E. et al. Silica crystals and aluminum salts regulate the production of prostaglandin in macrophages via NALP3 inflammasome-independent mechanisms. *Immunity* **34**, 514–526 (2011). 21497116 10.1016/j.immuni.2011.03.019

[21] Giamarellos-Bourboulis, E. J. et al. Crystals of monosodium urate monohydrate enhance lipopolysaccharide-induced release of interleukin 1β by mononuclear cells through a caspase 1-mediated process. *Ann. Rheum. Dis.* **68**, 273–278 (2009). 18390571 10.1136/ard.2007.082222

[22] Krantz, M., Eklund, D., Sarndahl, E. & Hedbrant, A. A detailed molecular network map and model of the NLRP3 inflammasome. *Front. Immunol.* **14**, 1233680 (2023). 38077364 10.3389/fimmu.2023.1233680 PMC10699087

[23] Munoz-Planillo, R. et al. K^+^ efflux is the common trigger of NLRP3 inflammasome activation by bacterial toxins and particulate matter. *Immunity* **38**, 1142–1153 (2013). 23809161 10.1016/j.immuni.2013.05.016 PMC3730833

[24] Di, A. et al. The TWIK2 potassium efflux channel in macrophages mediates NLRP3 inflammasome-induced inflammation. *Immunity* **49**, 56–65 (2018). 29958799 10.1016/j.immuni.2018.04.032 PMC6051907

[25] Huang, L. S. et al. Endosomal trafficking of two-pore K^+^ efflux channel TWIK2 to plasmalemma mediates NLRP3 inflammasome activation and inflammatory injury. *eL**ife*10.7554/eLife.83842 (2023). 10.7554/eLife.83842 PMC10202452 37158595

[26] Gross, C. J. et al. K^+^ efflux-independent NLRP3 inflammasome activation by small molecules targeting mitochondria. *Immunity* **45**, 761–773 (2016). 27692612 10.1016/j.immuni.2016.08.010

[27] Gaidt, M. M. et al. Human monocytes engage an alternative inflammasome pathway. *Immunity* **44**, 833–846 (2016). 27037191 10.1016/j.immuni.2016.01.012

[28] Schorn, C. et al. Sodium overload and water influx activate the NALP3 inflammasome. *J. Biol. Chem.* **286**, 35–41 (2011). 21051542 10.1074/jbc.M110.139048 PMC3012992

[29] Wang, J. et al. Hepatitis B virus-mediated sodium influx contributes to hepatic inflammation via synergism with intrahepatic danger signals. *iScience* **27**, 108723 (2024). 38283328 10.1016/j.isci.2023.108723 PMC10819783

[30] Tang, T. et al. CLICs-dependent chloride efflux is an essential and proximal upstream event for NLRP3 inflammasome activation. *Nat. Commun.* **8**, 202 (2017). 28779175 10.1038/s41467-017-00227-x PMC5544706

[31] Domingo-Fernandez, R., Coll, R. C., Kearney, J., Breit, S. & O’Neill, L. A. J. The intracellular chloride channel proteins CLIC1 and CLIC4 induce IL-1β transcription and activate the NLRP3 inflammasome. *J. Biol. Chem.* **292**, 12077–12087 (2017). 28576828 10.1074/jbc.M117.797126 PMC5519359

[32] Scambler, T. et al. ENaC-mediated sodium influx exacerbates NLRP3-dependent inflammation in cystic fibrosis. *eLife*10.7554/eLife.49248 (2019). 10.7554/eLife.49248 PMC6764826 31532390

[33] Mayes-Hopfinger, L. et al. Chloride sensing by WNK1 regulates NLRP3 inflammasome activation and pyroptosis. *Nat. Commun.* **12**, 4546 (2021). 34315884 10.1038/s41467-021-24784-4 PMC8316491

[34] Murakami, T. et al. Critical role for calcium mobilization in activation of the NLRP3 inflammasome. *Proc. Natl Acad. Sci. USA* **109**, 11282–11287 (2012). 22733741 10.1073/pnas.1117765109 PMC3396518

[35] Lee, G. S. et al. The calcium-sensing receptor regulates the NLRP3 inflammasome through Ca^2^^+^ and cAMP. *Nature* **492**, 123–127 (2012). 23143333 10.1038/nature11588 PMC4175565

[36] Zhou, R., Yazdi, A. S., Menu, P. & Tschopp, J. A role for mitochondria in NLRP3 inflammasome activation. *Nature* **469**, 221–225 (2011). 21124315 10.1038/nature09663

[37] Nakahira, K. et al. Autophagy proteins regulate innate immune responses by inhibiting the release of mitochondrial DNA mediated by the NALP3 inflammasome. *Nat. Immunol.* **12**, 222–230 (2011). 21151103 10.1038/ni.1980 PMC3079381

[38] Shimada, K. et al. Oxidized mitochondrial DNA activates the NLRP3 inflammasome during apoptosis. *Immunity* **36**, 401–414 (2012). 22342844 10.1016/j.immuni.2012.01.009 PMC3312986

[39] Zhong, Z. et al. New mitochondrial DNA synthesis enables NLRP3 inflammasome activation. *Nature* **560**, 198–203 (2018). 30046112 10.1038/s41586-018-0372-z PMC6329306

[40] Xian, H. et al. Metformin inhibition of mitochondrial ATP and DNA synthesis abrogates NLRP3 inflammasome activation and pulmonary inflammation. *Immunity* **54**, 1463–1477 (2021). 34115964 10.1016/j.immuni.2021.05.004 PMC8189765

[41] Billingham, L. K. et al. Mitochondrial electron transport chain is necessary for NLRP3 inflammasome activation. *Nat. Immunol.* **23**, 692–704 (2022). 35484407 10.1038/s41590-022-01185-3 PMC9098388

[42] Xiao, Y. et al. Mitochondrial dysfunction by FADDosome promotes gastric mucosal injury in portal hypertensive gastropathy. *Int. J. Biol. Sci.* **20**, 2658–2685 (2024). 38725851 10.7150/ijbs.90835 PMC11077381

[43] Subramanian, N., Natarajan, K., Clatworthy, M. R., Wang, Z. & Germain, R. N. The adaptor MAVS promotes NLRP3 mitochondrial localization and inflammasome activation. *Cell* **153**, 348–361 (2013). 23582325 10.1016/j.cell.2013.02.054 PMC3632354

[44] Iyer, S. S. et al. Mitochondrial cardiolipin is required for Nlrp3 inflammasome activation. *Immunity* **39**, 311–323 (2013). 23954133 10.1016/j.immuni.2013.08.001 PMC3779285

[45] Ichinohe, T., Yamazaki, T., Koshiba, T. & Yanagi, Y. Mitochondrial protein mitofusin 2 is required for NLRP3 inflammasome activation after RNA virus infection. *Proc. Natl Acad. Sci. USA* **110**, 17963–17968 (2013). 24127597 10.1073/pnas.1312571110 PMC3816452

[46] Martinon, F., Petrilli, V., Mayor, A., Tardivel, A. & Tschopp, J. Gout-associated uric acid crystals activate the NALP3 inflammasome. *Nature* **440**, 237–241 (2006). 16407889 10.1038/nature04516

[47] Dostert, C. et al. Innate immune activation through Nalp3 inflammasome sensing of asbestos and silica. *Science* **320**, 674–677 (2008). 18403674 10.1126/science.1156995 PMC2396588

[48] Halle, A. et al. The NALP3 inflammasome is involved in the innate immune response to amyloid-β. *Nat. Immunol.* **9**, 857–865 (2008). 18604209 10.1038/ni.1636 PMC3101478

[49] Duewell, P. et al. NLRP3 inflammasomes are required for atherogenesis and activated by cholesterol crystals. *Nature* **464**, 1357–1361 (2010). 20428172 10.1038/nature08938 PMC2946640

[50] Hou, Y., He, H., Ma, M. & Zhou, R. Apilimod activates the NLRP3 inflammasome through lysosome-mediated mitochondrial damage. *Front. Immunol.* **14**, 1128700 (2023). 37359517 10.3389/fimmu.2023.1128700 PMC10285205

[51] McCarthy, A. E., Yoshioka, C. & Mansoor, S. E. Full-Length P2X(7) structures reveal how palmitoylation prevents channel desensitization. *Cell* **179**, 659–670 (2019). 31587896 10.1016/j.cell.2019.09.017 PMC7053488

[52] Browne, L. E., Compan, V., Bragg, L. & North, R. A. P2X7 receptor channels allow direct permeation of nanometer-sized dyes. *J. Neurosci.* **33**, 3557–3566 (2013). 23426683 10.1523/JNEUROSCI.2235-12.2013 PMC6619550

[53] Wolf, A. J. et al. Hexokinase is an innate immune receptor for the detection of bacterial peptidoglycan. *Cell* **166**, 624–636 (2016). 27374331 10.1016/j.cell.2016.05.076 PMC5534359

[54] Xie, M. et al. PKM2-dependent glycolysis promotes NLRP3 and AIM2 inflammasome activation. *Nat. Commun.* **7**, 13280 (2016). 27779186 10.1038/ncomms13280 PMC5093342

[55] Tannahill, G. M. et al. Succinate is an inflammatory signal that induces IL-1β through HIF-1α. *Nature* **496**, 238–242 (2013). 23535595 10.1038/nature11986 PMC4031686

[56] Lampropoulou, V. et al. Itaconate links inhibition of succinate dehydrogenase with macrophage metabolic remodeling and regulation of inflammation. *Cell Metab.* **24**, 158–166 (2016). 27374498 10.1016/j.cmet.2016.06.004 PMC5108454

[57] Humphries, F. et al. Succination inactivates gasdermin D and blocks pyroptosis. *Science* **369**, 1633–1637 (2020). 32820063 10.1126/science.abb9818 PMC8744141

[58] Wen, H. et al. Fatty acid-induced NLRP3–ASC inflammasome activation interferes with insulin signaling. *Nat. Immunol.* **12**, 408–415 (2011). 21478880 10.1038/ni.2022 PMC4090391

[59] Xing, J. H. et al. NLRP3 inflammasome mediate palmitate-induced endothelial dysfunction. *Life Sci.* **239**, 116882 (2019). 31705915 10.1016/j.lfs.2019.116882

[60] Youm, Y. H. et al. The ketone metabolite beta-hydroxybutyrate blocks NLRP3 inflammasome-mediated inflammatory disease. *Nat. Med.* **21**, 263–269 (2015). 25686106 10.1038/nm.3804 PMC4352123

[61] Yan, H. et al. Hexokinase 2 senses fructose in tumor-associated macrophages to promote colorectal cancer growth. *Cell Metab.* **36**, 2449–2467 (2024). 39471815 10.1016/j.cmet.2024.10.002

[62] Misawa, T. et al. Microtubule-driven spatial arrangement of mitochondria promotes activation of the NLRP3 inflammasome. *Nat. Immunol.* **14**, 454–460 (2013). 23502856 10.1038/ni.2550

[63] Magupalli, V. G. et al. HDAC6 mediates an aggresome-like mechanism for NLRP3 and pyrin inflammasome activation. *Science*10.1126/science.aas8995 (2020). 10.1126/science.aas8995 PMC7814939 32943500

[64] Baldrighi, M. et al. PLK1 inhibition dampens NLRP3 inflammasome-elicited response in inflammatory disease models. *J. Clin. Invest.*10.1172/JCI162129 (2023). 10.1172/JCI162129 PMC10617773 37698938

[65] Guo, C. et al. Cholesterol homeostatic regulator SCAP–SREBP2 integrates NLRP3 inflammasome activation and cholesterol biosynthetic signaling in macrophages. *Immunity* **49**, 842–856 (2018). 30366764 10.1016/j.immuni.2018.08.021

[66] Chen, J. & Chen, Z. J. PtdIns4P on dispersed trans-Golgi network mediates NLRP3 inflammasome activation. *Nature* **564**, 71–76 (2018). 30487600 10.1038/s41586-018-0761-3 PMC9402428

[67] Nie, L. et al. Consecutive palmitoylation and phosphorylation orchestrates NLRP3 membrane trafficking and inflammasome activation. *Mol. Cell* **84**, 3336–3353 (2024). 39173637 10.1016/j.molcel.2024.08.001

[68] Yu, X. et al. Structural basis for the oligomerization-facilitated NLRP3 activation. *Nat. Commun.* **15**, 1164 (2024). 38326375 10.1038/s41467-024-45396-8 PMC10850481

[69] Schmacke, N. A. et al. IKKβ primes inflammasome formation by recruiting NLRP3 to the trans-Golgi network. *Immunity* **55**, 2271–2284 (2022). 36384135 10.1016/j.immuni.2022.10.021 PMC7614333

[70] Williams, D. M. & Peden, A. A. S-acylation of NLRP3 provides a nigericin sensitive gating mechanism that controls access to the Golgi. *eLife*10.7554/eLife.94302 (2024). 10.7554/eLife.94302 PMC11392533 39263961

[71] Ni, J. et al. Ubc13 Promotes K63-Linked Polyubiquitination of NLRP3 to Activate Inflammasome. *J. Immunol.* **206**, 2376–2385 (2021). 33893171 10.4049/jimmunol.2001178

[72] Tang, J. et al. Sequential ubiquitination of NLRP3 by RNF125 and Cbl-b limits inflammasome activation and endotoxemia. *J. Exp. Med.*10.1084/jem.20182091 (2020). 10.1084/jem.20182091 PMC7144527 31999304

[73] Song, H. et al. The E3 ubiquitin ligase TRIM31 attenuates NLRP3 inflammasome activation by promoting proteasomal degradation of NLRP3. *Nat. Commun.* **7**, 13727 (2016). 27929086 10.1038/ncomms13727 PMC5155141

[74] Li, Y. T. et al. USP13 stabilizes NLRP3 to facilitate inflammasome activation by preventing TRIM31-mediated NLRP3 ubiquitination and degradation. *Sci. Adv.* **11**, eadx3827 (2025). 41004574 10.1126/sciadv.adx3827 PMC12466843

[75] Yan, Y. et al. Dopamine controls systemic inflammation through inhibition of NLRP3 inflammasome. *Cell* **160**, 62–73 (2015). 25594175 10.1016/j.cell.2014.11.047

[76] Wan, P. et al. Cullin1 binds and promotes NLRP3 ubiquitination to repress systematic inflammasome activation. *FASEB J.* **33**, 5793–5807 (2019). 30653357 10.1096/fj.201801681R

[77] Han, S. et al. Lipopolysaccharide primes the NALP3 inflammasome by inhibiting its ubiquitination and degradation mediated by the SCFFBXL2 E3 ligase. *J. Biol. Chem.* **290**, 18124–18133 (2015). 26037928 10.1074/jbc.M115.645549 PMC4505057

[78] Kawashima, A. et al. ARIH2 ubiquitinates NLRP3 and negatively regulates NLRP3 inflammasome activation in macrophages. *J. Immunol.* **199**, 3614–3622 (2017). 29021376 10.4049/jimmunol.1700184

[79] Xu, T. et al. Ubiquitination of NLRP3 by gp78/Insig-1 restrains NLRP3 inflammasome activation. *Cell Death Differ.* **29**, 1582–1595 (2022). 35110683 10.1038/s41418-022-00947-8 PMC9345978

[80] Ge, X. et al. PARK2 attenuates house dust mite-induced inflammatory reaction, pyroptosis and barrier dysfunction in BEAS-2B cells by ubiquitinating NLRP3. *Am. J. Transl. Res.* **13**, 326–335 (2021). 33527027 PMC7847526

[81] Park, Y. J. et al. MARCH5-dependent NLRP3 ubiquitination is required for mitochondrial NLRP3–NEK7 complex formation and NLRP3 inflammasome activation. *EMBO J.* **42**, e113481 (2023). 37575012 10.15252/embj.2023113481 PMC10548170

[82] Tang, T. et al. The E3 ubiquitin ligase TRIM65 negatively regulates inflammasome activation through promoting ubiquitination of NLRP3. *Front. Immunol.* **12**, 741839 (2021). 34512673 10.3389/fimmu.2021.741839 PMC8427430

[83] Guo, Y. et al. HUWE1 mediates inflammasome activation and promotes host defense against bacterial infection. *J. Clin. Invest.* **130**, 6301–6316 (2020). 33104527 10.1172/JCI138234 PMC7685759

[84] Wang, D. et al. YAP promotes the activation of NLRP3 inflammasome via blocking K27-linked polyubiquitination of NLRP3. *Nat. Commun.* **12**, 2674 (2021). 33976226 10.1038/s41467-021-22987-3 PMC8113592

[85] Humphries, F. et al. The E3 ubiquitin ligase Pellino2 mediates priming of the NLRP3 inflammasome. *Nat. Commun.* **9**, 1560 (2018). 29674674 10.1038/s41467-018-03669-z PMC5908787

[86] Hang, Y., Tan, L., Chen, Q., Liu, Q. & Jin, Y. E3 ubiquitin ligase TRIM24 deficiency promotes NLRP3/caspase-1/IL-1β-mediated pyroptosis in endometriosis. *Cell Biol. Int.* **45**, 1561–1570 (2021). 33724611 10.1002/cbin.11592

[87] Shen, J. et al. TRIM40 inhibits IgA1-induced proliferation of glomerular mesangial cells by inactivating NLRP3 inflammasome through ubiquitination. *Mol. Immunol.* **140**, 225–232 (2021). 34763147 10.1016/j.molimm.2021.10.012

[88] Lin, Y. et al. TRIM50 promotes NLRP3 inflammasome activation by directly inducing NLRP3 oligomerization. *EMBO Rep.* **23**, e54569 (2022). 36178239 10.15252/embr.202154569 PMC9638864

[89] Song, H. et al. UAF1 deubiquitinase complexes facilitate NLRP3 inflammasome activation by promoting NLRP3 expression. *Nat. Commun.* **11**, 6042 (2020). 33247121 10.1038/s41467-020-19939-8 PMC7695691

[90] Li, X. et al. MF-094, a potent and selective USP30 inhibitor, accelerates diabetic wound healing by inhibiting the NLRP3 inflammasome. *Exp. Cell Res.* **410**, 112967 (2022). 34883112 10.1016/j.yexcr.2021.112967

[91] Zhao, C. et al. USP50 regulates NLRP3 inflammasome activation in duodenogastric reflux-induced gastric tumorigenesis. *Front. Immunol.* **15**, 1326137 (2024). 38469295 10.3389/fimmu.2024.1326137 PMC10925683

[92] Xiang, Y., Li, X., Cai, M. & Cai, D. USP9X promotes lipopolysaccharide-stimulated acute lung injury by deubiquitination of NLRP3. *Cell Biol. Int.* **47**, 394–405 (2023). 36525374 10.1002/cbin.11932

[93] Lu, H. et al. The E3 ubiquitin ligase MARCH9 alleviates pyroptosis by regulating NLPR3 ubiquitination following myocardial ischemia reperfusion. *Cell. Mol. Life Sci.* **82**, 348 (2025). 41055760 10.1007/s00018-025-05861-z PMC12504159

[94] Wang, W. et al. STING promotes NLRP3 localization in ER and facilitates NLRP3 deubiquitination to activate the inflammasome upon HSV-1 infection. *PLoS Pathog.* **16**, e1008335 (2020). 32187211 10.1371/journal.ppat.1008335 PMC7080238

[95] Zhang, W. et al. Cytosolic escape of mitochondrial DNA triggers cGAS–STING–NLRP3 axis-dependent nucleus pulposus cell pyroptosis. *Exp. Mol. Med.* **54**, 129–142 (2022). 35145201 10.1038/s12276-022-00729-9 PMC8894389

[96] Py, B. F., Kim, M. S., Vakifahmetoglu-Norberg, H. & Yuan, J. Deubiquitination of NLRP3 by BRCC3 critically regulates inflammasome activity. *Mol. Cell* **49**, 331–338 (2013). 23246432 10.1016/j.molcel.2012.11.009

[97] Ren, G. et al. ABRO1 promotes NLRP3 inflammasome activation through regulation of NLRP3 deubiquitination. *EMBO J.*10.15252/embj.2018100376 (2019). 10.15252/embj.2018100376 PMC6418445 30787184

[98] Yalcinkaya, M. et al. BRCC3-mediated NLRP3 deubiquitylation promotes inflammasome activation and atherosclerosis in Tet2 clonal hematopoiesis. *Circulation* **148**, 1764–1777 (2023). 37781816 10.1161/CIRCULATIONAHA.123.065344 PMC10872582

[99] Liu, C. et al. YOD1 protects against MRSA sepsis-induced DIC through Lys33-linked deubiquitination of NLRP3. *Cell Death Dis.* **15**, 360 (2024). 38789414 10.1038/s41419-024-06731-5 PMC11126606

[100] Palazon-Riquelme, P. et al. USP7 and USP47 deubiquitinases regulate NLRP3 inflammasome activation. *EMBO Rep.*10.15252/embr.201744766 (2018). 10.15252/embr.201744766 PMC6172458 30206189

[101] Rao, Z. et al. Vitamin D receptor inhibits NLRP3 activation by impeding its BRCC3-mediated deubiquitination. *Front. Immunol.* **10**, 2783 (2019). 31866999 10.3389/fimmu.2019.02783 PMC6904361

[102] Bednash, J. S. et al. The deubiquitinase STAMBP modulates cytokine secretion through the NLRP3 inflammasome. *Cell. Signal.* **79**, 109859 (2021). 33253913 10.1016/j.cellsig.2020.109859 PMC10201604

[103] Zhang, Z. et al. Protein kinase D at the Golgi controls NLRP3 inflammasome activation. *J. Exp. Med.* **214**, 2671–2693 (2017). 28716882 10.1084/jem.20162040 PMC5584123

[104] Mortimer, L., Moreau, F., MacDonald, J. A. & Chadee, K. NLRP3 inflammasome inhibition is disrupted in a group of auto-inflammatory disease CAPS mutations. *Nat. Immunol.* **17**, 1176–1186 (2016). 27548431 10.1038/ni.3538

[105] Guo, C. et al. Bile acids control inflammation and metabolic disorder through inhibition of NLRP3 inflammasome. *Immunity* **45**, 802–816 (2016). 27692610 10.1016/j.immuni.2016.09.008

[106] Guo, X. et al. AKT controls NLRP3 inflammasome activation by inducing DDX3X phosphorylation. *FEBS Lett.* **595**, 2447–2462 (2021). 34387860 10.1002/1873-3468.14175

[107] Zhu, K. et al. Priming of NLRP3 inflammasome activation by Msn kinase MINK1 in macrophages. *Cell. Mol. Immunol.* **18**, 2372–2382 (2021). 34480147 10.1038/s41423-021-00761-1 PMC8414466

[108] Chung, I. C. et al. Pyk2 activates the NLRP3 inflammasome by directly phosphorylating ASC and contributes to inflammasome-dependent peritonitis. *Sci. Rep.* **6**, 36214 (2016). 27796369 10.1038/srep36214 PMC5087076

[109] Niu, T. et al. NLRP3 phosphorylation in its LRR domain critically regulates inflammasome assembly. *Nat. Commun.* **12**, 5862 (2021). 34615873 10.1038/s41467-021-26142-w PMC8494922

[110] Ma, Z. et al. PGK1 phosphorylates NLRP3 and mediates inflammasome activation independent of its glycolytic activity. *Cell Rep.* **44**, 115785 (2025). 40471786 10.1016/j.celrep.2025.115785

[111] Kim, H. J. et al. The Src family kinase, Lyn, suppresses osteoclastogenesis in vitro and in vivo. *Proc. Natl Acad. Sci. USA* **106**, 2325–2330 (2009). 19171907 10.1073/pnas.0806963106 PMC2650155

[112] Bittner, Z. A. et al. BTK operates a phospho-tyrosine switch to regulate NLRP3 inflammasome activity. *J. Exp. Med.*10.1084/jem.20201656 (2021). 10.1084/jem.20201656 PMC8480672 34554188

[113] Zhang, A. et al. EphA2 phosphorylates NLRP3 and inhibits inflammasomes in airway epithelial cells. *EMBO Rep.* **21**, e49666 (2020). 32352641 10.15252/embr.201949666 PMC7332978

[114] Spalinger, M. R. et al. PTPN22 regulates NLRP3-mediated IL1B secretion in an autophagy-dependent manner. *Autophagy* **13**, 1590–1601 (2017). 28786745 10.1080/15548627.2017.1341453 PMC5612532

[115] Spalinger, M. R. et al. NLRP3 tyrosine phosphorylation is controlled by protein tyrosine phosphatase PTPN22. *J. Clin. Invest.*10.1172/JCI169304 (2023). 10.1172/JCI169304 PMC9927928 36787260

[116] Zhao, W. et al. AKT regulates NLRP3 inflammasome activation by phosphorylating NLRP3 serine 5. *J. Immunol.* **205**, 2255–2264 (2020). 32929041 10.4049/jimmunol.2000649 PMC7541779

[117] Fischer, F. A. et al. TBK1 and IKKepsilon act like an OFF switch to limit NLRP3 inflammasome pathway activation. *Proc. Natl Acad. Sci. USA*10.1073/pnas.2009309118 (2021). 10.1073/pnas.2009309118 PMC8463895 34518217

[118] Zhang, Y. et al. Acetylation is required for full activation of the NLRP3 inflammasome. *Nat. Commun.* **14**, 8396 (2023). 38110429 10.1038/s41467-023-44203-0 PMC10728138

[119] He, M. et al. An acetylation switch of the NLRP3 inflammasome regulates aging-associated chronic inflammation and insulin resistance. *Cell Metab.* **31**, 580–591 (2020). 32032542 10.1016/j.cmet.2020.01.009 PMC7104778

[120] Zhang, L. et al. Tau induces inflammasome activation and microgliosis through acetylating NLRP3. *Clin. Transl. Med.* **14**, e1623 (2024). 38488468 10.1002/ctm2.1623 PMC10941548

[121] Guan, X. et al. Inhibition of HDAC2 sensitises antitumour therapy by promoting NLRP3/GSDMD-mediated pyroptosis in colorectal cancer. *Clin. Transl. Med.* **14**, e1692 (2024). 38804602 10.1002/ctm2.1692 PMC11131357

[122] Lin, Q. C. et al. Hippocampal HDAC6 promotes POCD by regulating NLRP3-induced microglia pyroptosis via HSP90/HSP70 in aged mice. *Biochim. Biophys. Acta Mol. Basis Dis.* **1870**, 167137 (2024). 38527593 10.1016/j.bbadis.2024.167137

[123] Yang, M. et al. HDAC10 switches NLRP3 modification from acetylation to ubiquitination and attenuates acute inflammatory diseases. *Cell Commun. Signal.* **22**, 615 (2024). 39707387 10.1186/s12964-024-01992-1 PMC11662490

[124] Shao, L. et al. SUMO1 SUMOylates and SENP3 deSUMOylates NLRP3 to orchestrate the inflammasome activation. *FASEB J.* **34**, 1497–1515 (2020). 31914638 10.1096/fj.201901653R

[125] Barry, R. et al. SUMO-mediated regulation of NLRP3 modulates inflammasome activity. *Nat. Commun.* **9**, 3001 (2018). 30069026 10.1038/s41467-018-05321-2 PMC6070540

[126] Chen, J. et al. SENP3 attenuates foam cell formation by deSUMOylating NLRP3 in macrophages stimulated with ox-LDL. *Cell. Signal.* **117**, 111092 (2024). 38331013 10.1016/j.cellsig.2024.111092

[127] Jing, F. et al. Zmiz1-mediated SUMOylation of NLRP3 inflammasome regulates satellite glial cell activation and neuronal autophagy in trigeminal neuralgia. *Inflammation* **48**, 4342–4363 (2025). 40650830 10.1007/s10753-025-02330-4 PMC12722413

[128] Qin, Y. et al. TRIM28 SUMOylates and stabilizes NLRP3 to facilitate inflammasome activation. *Nat. Commun.* **12**, 4794 (2021). 34373456 10.1038/s41467-021-25033-4 PMC8352945

[129] Iarmonenko, S. P. Radiomodifiers and the progress of radiation oncology]. *Vopr. Onkol.* **41**, 93–94 (1995). 7483456

[130] Shim, D. W. et al. BOT-4-one attenuates NLRP3 inflammasome activation: NLRP3 alkylation leading to the regulation of its ATPase activity and ubiquitination. *Sci. Rep.* **7**, 15020 (2017). 29118366 10.1038/s41598-017-15314-8 PMC5678161

[131] Juliana, C. et al. Anti-inflammatory compounds parthenolide and Bay 11-7082 are direct inhibitors of the inflammasome. *J. Biol. Chem.* **285**, 9792–9802 (2010). 20093358 10.1074/jbc.M109.082305 PMC2843228

[132] He, Y. et al. 3,4-Methylenedioxy-β-nitrostyrene inhibits NLRP3 inflammasome activation by blocking assembly of the inflammasome. *J. Biol. Chem.* **289**, 1142–1150 (2014). 24265316 10.1074/jbc.M113.515080 PMC3887181

[133] Yang, S. et al. ABHD8 antagonizes inflammation by facilitating chaperone-mediated autophagy-mediated degradation of NLRP3. *Autophagy* **21**, 338–351 (2025). 39225180 10.1080/15548627.2024.2395158 PMC11759624

[134] Zheng, S. et al. ZDHHC5-mediated NLRP3 palmitoylation promotes NLRP3–NEK7 interaction and inflammasome activation. *Mol. Cell* **83**, 4570–4585.e4577 (2023). 38092000 10.1016/j.molcel.2023.11.015

[135] Hu, D. et al. Palmitoylation of NLRP3 modulates inflammasome activation and inflammatory bowel disease development. *J. Immunol.* **213**, 481–493 (2024). 38949555 10.4049/jimmunol.2300241 PMC11299489

[136] Zou, G. et al. Signal-induced NLRP3 phase separation initiates inflammasome activation. *Cell Res.* **35**, 437–452 (2025). 40164768 10.1038/s41422-025-01096-6 PMC12134225

[137] Chiu, L. Y., Huang, D. Y. & Lin, W. W. PARP-1 regulates inflammasome activity by poly-ADP-ribosylation of NLRP3 and interaction with TXNIP in primary macrophages. *Cell. Mol. Life. Sci.* **79**, 108 (2022). 35098371 10.1007/s00018-022-04138-z PMC8801414

[138] Bose, S. et al. ADP-ribosylation of NLRP3 by *Mycoplasma pneumoniae* CARDS toxin regulates inflammasome activity. *mBio*10.1128/mBio.02186-14 (2014). 10.1128/mBio.02186-14 PMC4278538 25538194

[139] Luo, L. et al. STAT3 promotes NLRP3 inflammasome activation by mediating NLRP3 mitochondrial translocation. *Exp. Mol. Med.* **56**, 1980–1990 (2024). 39218978 10.1038/s12276-024-01298-9 PMC11446920

[140] Liang, Z. et al. Proximity proteomics reveals UCH-L1 as an essential regulator of NLRP3-mediated IL-1β production in human macrophages and microglia. *Cell Rep.* **43**, 114152 (2024). 38669140 10.1016/j.celrep.2024.114152

[141] Ha, J. et al. SERTAD1 initiates NLRP3-mediated inflammasome activation through restricting NLRP3 polyubiquitination. *Cell Rep.* **43**, 113752 (2024). 38341852 10.1016/j.celrep.2024.113752

[142] Yu, J. et al. Inhibition of NLRP3 inflammasome activation by A20 through modulation of NEK7. *Proc. Natl Acad. Sci. USA* **121**, e2316551121 (2024). 38865260 10.1073/pnas.2316551121 PMC11194493

[143] Guo, X. B. et al. HAX-1 interferes in assembly of NLRP3–ASC to block microglial pyroptosis in cerebral I/R injury. *Cell Death Discov.* **10**, 264 (2024). 38811533 10.1038/s41420-024-02005-3 PMC11136987

[144] Liu, S. et al. The E3 ubiquitin ligase MARCH2 protects against myocardial ischemia–reperfusion injury through inhibiting pyroptosis via negative regulation of PGAM5/MAVS/NLRP3 axis. *Cell Discov.* **10**, 24 (2024). 38409220 10.1038/s41421-023-00622-3 PMC10897310

[145] Huang, J. et al. The human disease-associated gene ZNFX1 controls inflammation through inhibition of the NLRP3 inflammasome. *EMBO J.* **43**, 5469–5493 (2024). 39333773 10.1038/s44318-024-00236-9 PMC11574294

[146] Yang, S. et al. ABHD8 antagonizes inflammation by facilitating chaperone-mediated autophagy-mediated degradation of NLRP3. *Autophagy* **21**, 1–14 (2024). 10.1080/15548627.2024.2395158 PMC11759624 39225180

[147] Cheng, Z. et al. HECTD3 inhibits NLRP3 inflammasome assembly and activation by blocking NLRP3–NEK7 interaction. *Cell Death Dis.* **15**, 86 (2024). 38267403 10.1038/s41419-024-06473-4 PMC10808187

